# Conservative management of knee arthropathy in a patient with Klippel Trenaunay syndrome

**DOI:** 10.1590/1677-5449.200010

**Published:** 2020-05-20

**Authors:** Fanny Rodríguez Santos, Victoria Loson, Agustín Coria, Hugo Martínez

**Affiliations:** 1 Hospital Italiano de Buenos Aires, General Surgery Department, Phlebolymphology Unit, Buenos Aires, Argentina.

**Keywords:** Klippel-Trenaunay syndrome, vascular malformation, knee arthropathy, síndrome de Klippel-Trenaunay, malformação vascular, artropatia do joelho

## Abstract

Klippel-Trenaunay syndrome (KTS) is a rare vascular malformation characterized by capillary malformation, venous malformations, and soft tissue or bone hypertrophy that affect the extremities in most cases. Knee or hip arthropathy are common associated conditions and cause serious disability. We present the case of a patient with a diagnosis of KTS and severe knee arthropathy. A 34-year-old man with KTS was referred to our hospital with severe knee arthropathy, with the joint fixed in a 90° position. CT Angiography and MRI of the left leg showed important varicose development of the superficial venous system with intraarticular vessels. After discussion of the case by a multidisciplinary committee, the patient was enrolled on a physiotherapy program and had achieved significant improvements in movement and quality of life at 12-month follow-up. Treatment of KTS is primarily conservative and a multidisciplinary approach is necessary.

## Resumo

A síndrome de Klippel-Trenaunay (SKT) é uma malformação vascular rara caracterizada por malformação capilar, malformações venosas e hipertrofia de tecidos moles ou ósseos que afetam as extremidades na maioria dos casos. A artropatia do joelho ou do quadril é uma condição comumente associada e causa sérias deficiências. Apresentamos o caso de um paciente com diagnóstico de SKT e artropatia grave do joelho. Um homem de 34 anos com SKT foi encaminhado ao nosso hospital com artropatia grave do joelho com articulação fixa na posição de 90 °. A angiotomografia e a ressonância magnética da perna esquerda mostraram importante desenvolvimento varicoso do sistema venoso superficial com vasos intra-articulares. Após o caso ser discutido em um comitê multidisciplinar, o paciente foi incluído em um programa de fisioterapia, obtendo uma melhora significativa nos movimentos e na qualidade de vida após 12 meses de acompanhamento. O tratamento da SKT é principalmente conservador e exige uma abordagem multidisciplinar.

## INTRODUCTION

Klippel Trenaunay syndrome (KTS) is a rare complex vascular malformation characterized by three clinical features including capillary malformation (port-wine stain), venous malformations, and soft tissue or bone hypertrophy, in most cases involving the extremities. KTS mainly occurs sporadically with only rare cases of family history and its etiology has not yet been clarified.^1^

There is a broad spectrum of clinical manifestations, attributed to the unpredictable nature of vascular malformations and their complications, including cellulitis, lymphedema, or deep vein thrombosis, and occasionally hematuria or hematochezia, when an internal organ is affected. Although the course of KTS is mostly benign, patients are at higher risk of developing thromboembolism and life-threatening hemorrhages.^2^

Knee or hip arthropathy and disparity in leg lengths are common associated conditions that cause severe disability. We describe the conservative management of a 34-year-old patient with a diagnosis of KTS and severe knee arthropathy. The patient consented to publication of this report.

### Case report

A 34-year-old man with KTS and no surgical history was referred to our hospital with severe knee arthropathy that had been worsening over the preceding months. He mentioned stiffness of the left knee and inability to perform flexion-extension movements, to the point of being unable to walk on his own, and rated the pain as 10 out of 10 with poor response to analgesics.

**Figure 1:**
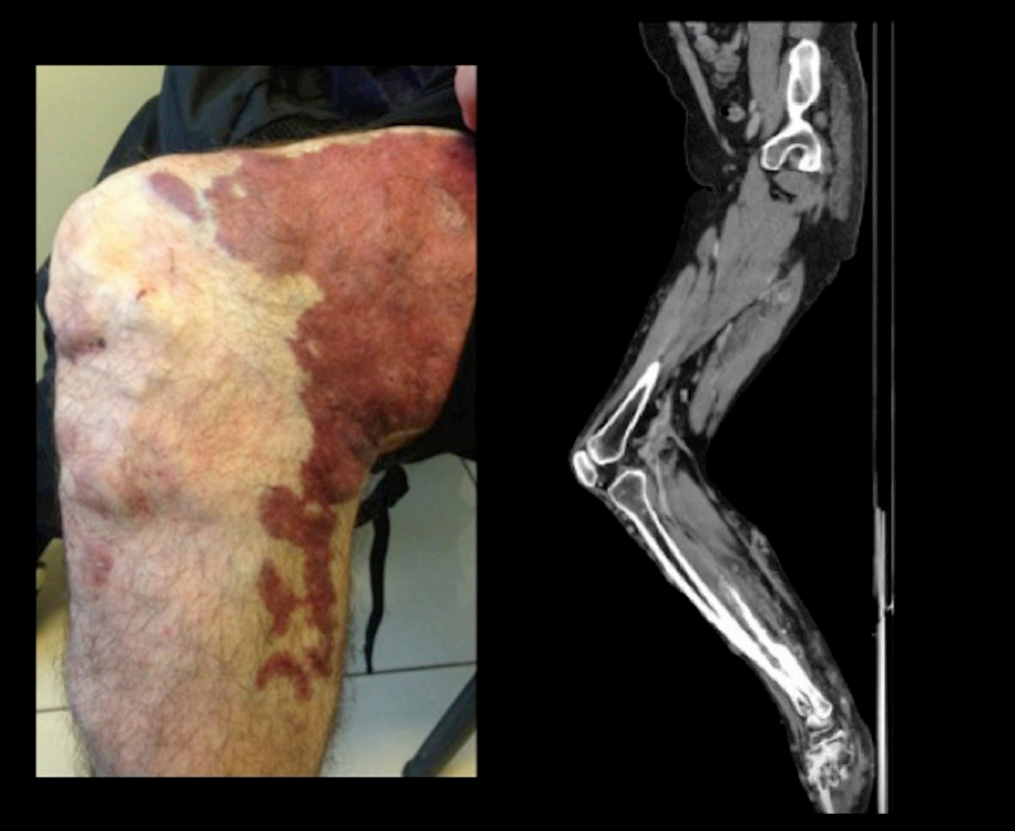
Extensive palpable varicose veins and changes to the skin of the left leg. The fixed flexion position of the knee joint is also evidenced by CT Angiography.

His physical examination was remarkable for the significantly larger diameter of the left leg, with extensive palpable varicose veins and changes to the skin consistent with venous stasis. The position of his knee joint was fixed, with movement restricted from 75 degrees in extension to 90 degrees in flexion (Figure 1). Discrepancies in leg length and diameter were difficult to measure due to flexion contracture. Neurologic findings were normal and while the dorsalis pedis and posterior tibial arteries were difficult to palpate, the extremity was well perfused.

**Figure 2:**
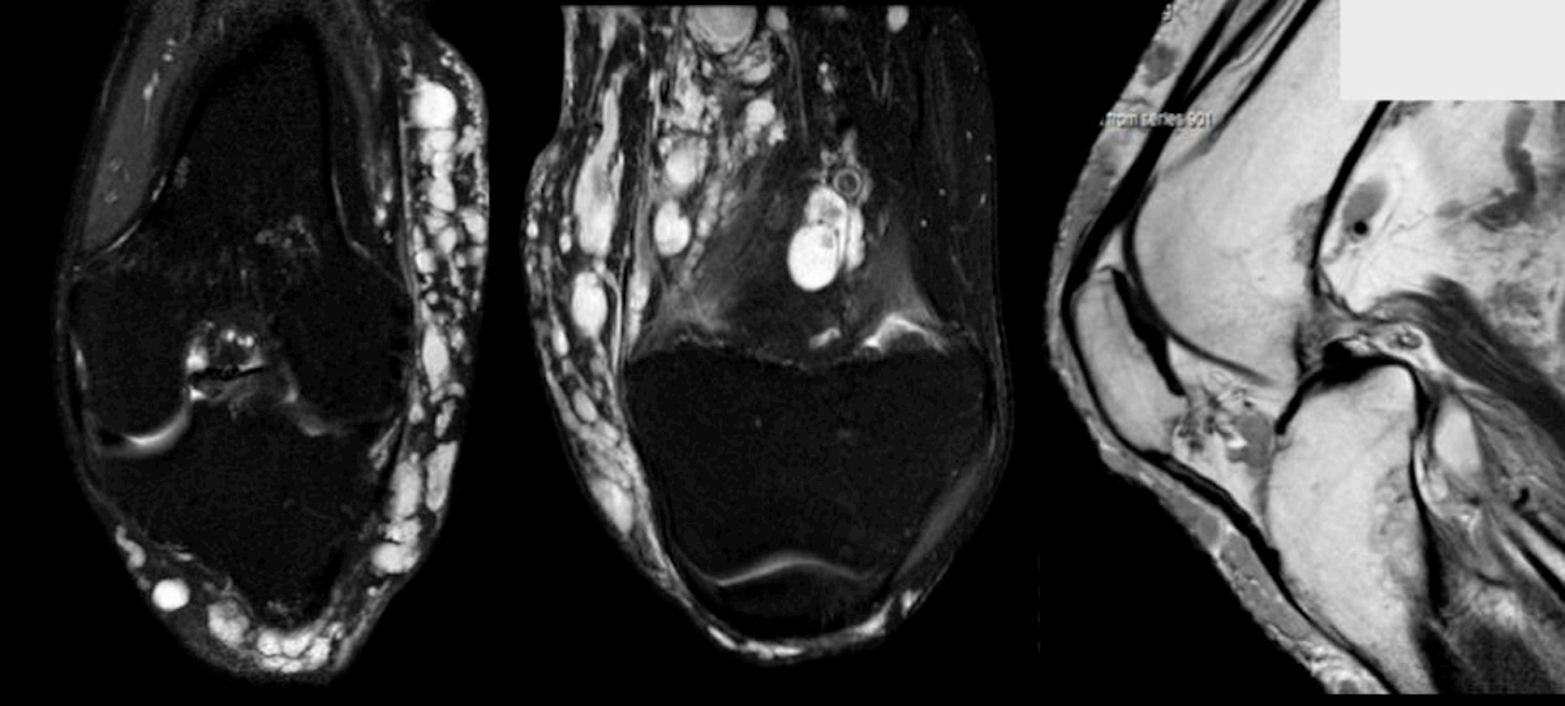
MRI shows important varicose development involving the superficial venous system, with intraarticular and intramuscular vessels, increased soft tissue component and bone hypertrophy, but no alterations in joint structures.

A Doppler ultrasound examination (DUS) showed absence of venous obstruction and normal arterial flow. CT Angiography of the leg evidenced important varicose development of the superficial venous system, increased soft tissue component, and bone hypertrophy with marked thickening and cortical irregularity in the fibula and the distal part of the femur. Magnetic resonance imaging (MRI) showed intraarticular varicose vessels as well as intramuscular location in the biceps femoris and semimembranosus and involvement of sciatic nerves, with no alterations of joint structures (Figure 2).

The case was presented to a multidisciplinary committee including vascular and orthopedic surgeons. Based on the benign prognosis and the high morbidity of surgical treatment, conservative management was chosen as the first option. The patient was enrolled on a physiotherapy program, which consisted of attending a rehabilitation clinic, twice a week initially and then twice a month after the ninth month of treatment, where he performed strengthening and functional exercises to improve range of motion and joint stability, in combination with passive manual mobilization. In association, weekly manual lymphatic drainage was indicated during the initial months to reduce phleboedema secondary to venous stasis and he was prescribed elastic bandages and venotonic medication. Once a satisfactory reduction in the diameter of the leg had been obtained, a 20-30 mmHg compression stocking was indicated.

**Figure 3:**
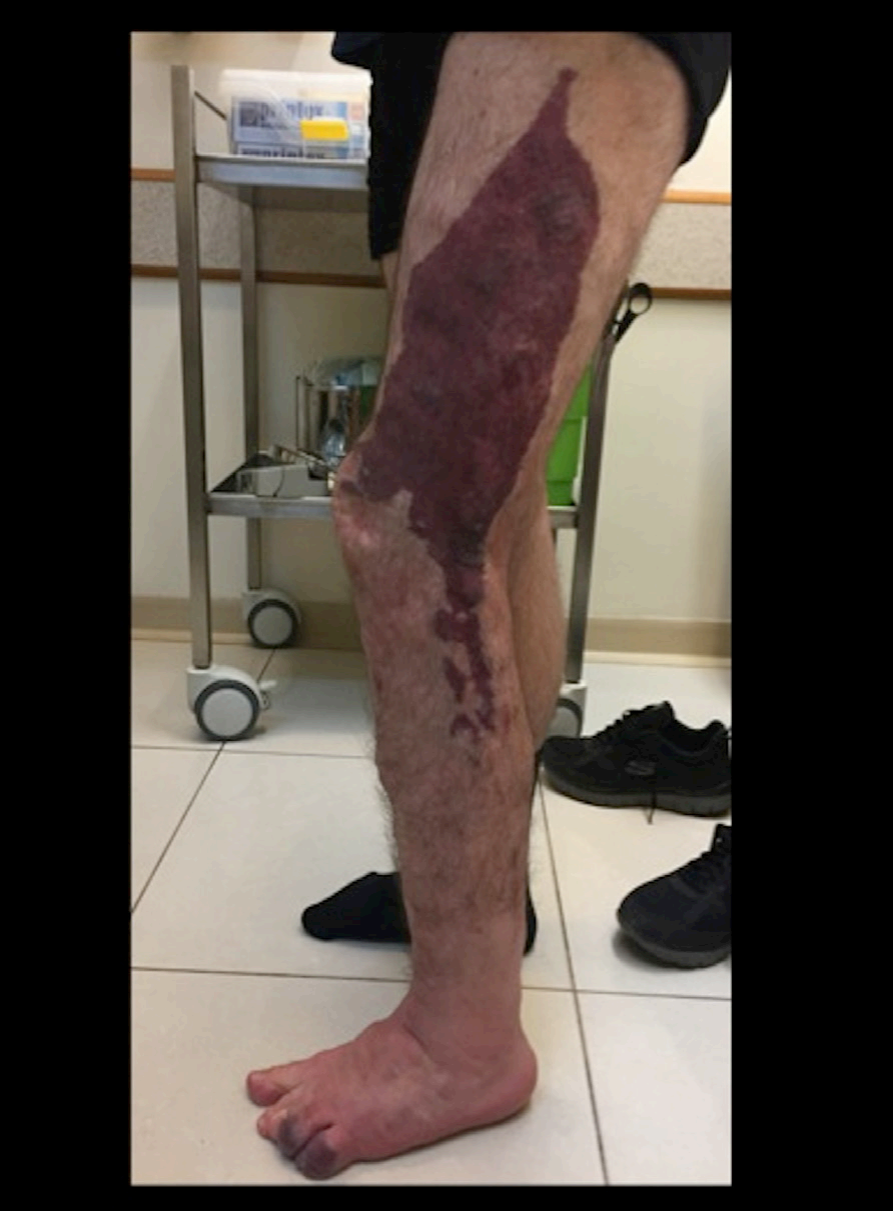
Significant improvement in the range of motion after 12 months of conservative multidisciplinary treatment.

At 12 months of follow-up, significant improvement in his range of motion had been achieved, from 10 degrees in extension to 100 degrees in flexion, which allowed him to stand for several hours and walk without external assistance (crutches) and improved his quality of life (Figure 3). Future follow-up will define whether a surgical approach is necessary or not.

## DISCUSSION

Diagnosis of KTS is mainly clinical and the physical examination is commonly complemented with DUS to establish patency and competence of the venous system and detect an arteriovenous shunt if present.

KTS is a chronic disease and, although the course is benign in most cases, symptoms and complications can severely affect quality of life.

Venous malformations in the compromised limb can cause pain, edema, thrombophlebitis, ulcers, and bleeding. Additionally, vascular malformations can lead to bone and soft-tissue hypertrophy causing serious disability due to knee or hip arthropathy and disparity in leg lengths. Abnormalities of the lymphatic system and chronic venous insufficiency can lead to lymphedema and when internal organs are affected, symptoms like hematuria and hematochezia can appear. If a high-flow arteriovenous fistula is present, the presentation is called Parkes-Weber Syndrome and prognosis is worse.

On the other hand, patients with KTS are at risk of thromboembolic disease. Baskerville et al. reported 14% of pulmonary embolism and 16% of DVT in 46 patients.^3^^,^^4^

Treatment of KTS is primarily conservative and a multidisciplinary approach is required to provide optimal care tailored to each patient.

In case of symptomatic lymphedema, a combination of therapies including compression therapy, manual lymph drainage, intermittent pneumatic compression, and hygiene care is recommended.^5^ In patients with associated arthropathy, physiotherapy interventions such as exercises and manual mobilization techniques can reduce knee pain and improve function.^6^

Medications such as anticoagulant, venotonic, lymphokinetic, and anti-inflammatory drugs may be necessary.^7^^,^^8^ Psychological support of the patient and family is also important.^1^

Surgical interventions are reserved selectively for patients refractory to conservative management or when complications occur. They include minimally invasive procedures such as sclerotherapy, thermal ablations, and embolizations, open surgery consisting of vein stripping or stab phlebectomies, and orthopedic procedures.^2^

Sung et al. described the clinical management of 19 patients with KTS. In 4.1 years of follow up, only 4 patients required interventions: 3 treated with sclerotherapy and 1 with vein ligation and stripping.^9^

If indicated, surgery must be preceded by careful evaluation of the extent of malformations and patency of the deep venous system with a CT Scan or venography. Simple X-rays are used to measure bone length and magnetic resonance imaging (MRI) is used to assess involvement of fat, joints, and muscles.

With respect to knee arthropathy associated with KTS, there are cases reported in the literature that were treated with surgical procedures with good results (Table 1). However, these procedures were only indicated in refractory cases and the risks of the procedures were considered, such as wound complications, postoperative anemia, cardiovascular complications, infections, and thromboembolic events.^10^^-^15

**Table 1:** Surgical treatment for Knee Arthropathy associated with Klippel-Trenaunay Syndrome: Cases reported in the Literature.

**Author**	**Publication**	**n**	**Age**	**Gender**	**Indication**	**Procedure**	**Previous treatment**	**Complications**	**FU (m)** *	**Improved** †
Joseph et al.^10^	JBJS Case Connect. 2017	1	66	M	pain, motion, infection	TKA	conservative	BCT heart attack hemorrhage: surgery	26	yes
Bhende et al.^11^	Indian J Orthop. 2015	1	30	F	Pain	navigated TKA	conservative	BCT	12	yes
Leal et al.^12^	J. Arthroplasty. 2008	1	38	M	pain, motion	TKA	synovectomy	none	60	yes
Catre et al.^13^	Can J Sur. 2005	1	35	M	pain, motion	TKA	conservative	none	-	yes
Johnson et al.^14^	J Pediatr Orthop. 2009	7	13 (5-23)	5 M	pain, motion, infection	4 synovectomy 4 knee disarticulation	conservative	4 BCT, 2 wound dehiscence 1 hemorrhage, DIC	73.1 (7-109)	yes
				2 F						
Labott et al.^15^	J. Arthroplasty. 2019	12	39 (22-61)	6 M	pain, motion	TKA	1 synovectomy1 arthroscopy	BCT 1 infection: surgery	84 (2-204)	yes
				6 F			2 meniscectomy	1 bone loss: surgery		
							1 epiphysiodesis 6 conservative			

n = number of cases reported; M = male; F = female; BCT = Blood Cells transfusion; DIC = Disseminated intravascular coagulation; TKA = Total Knee Arthroplasty.

* FU (m) follow up in months;

† Improvement after procedure.

## CONCLUSION

Knee arthropathy is a condition commonly associated with KTS. While surgical treatments have been reported with good results, conservative management remains the first option.

Decision-making should be multidisciplinary and based on the symptoms and prognosis of each patient.
